# Integrated Pharmacoepigenomic Analysis Uncovers the Impact of Antiseizure Medications on Developmental Pathways and the Protective Effect of Folic Acid

**DOI:** 10.3390/ijms26167981

**Published:** 2025-08-19

**Authors:** Neethu Mohan, Moinak Banerjee

**Affiliations:** 1 Human Molecular Genetics Lab, Neurobiology and Genetics Division, BRIC-Rajiv Gandhi Centre for Biotechnology, Thiruvananthapuram 695014, India; neethumohan@rgcb.res.in; 2 BRIC-Regional Centre for Biotechnology, NCR Biotech Science Cluster, Faridabad 121001, India

**Keywords:** antiseizure medication, teratogen, DNA methylation, genome-wide methylation, proteomics, biomarker, pregnancy, precision medicine, pharmacoepigenomics

## Abstract

Fetal exposure to antiseizure medications (ASMs) can impact organogenesis, resulting in elevated risk of congenital malformations. Despite longstanding clinical awareness of the teratogenic potential of ASMs, the molecular mechanisms remain largely unexplored. To address this multisystem impact of ASMs, an OMIC-based approach was considered to understand the impact of ASMs on methylome and subsequently on proteome and how folic acid (FA) supplementation can counter the teratogenic impact. The study employed an established in vitro embryonic cell line model system, treated with varying concentrations of first-generation ASMs, alone and in combination with FA. Integrated analyses included quantification of global DNA methylation, expression analysis of key epigenetic regulators (DNMTs and TETs), genome-wide methylation profiling using the 935K EPIC array, and LC-MS/MS-based proteomics analysis. The study identified that ASMs can induce global DNA hypomethylation, which was likely to be impacted by dysregulation of DNMT and TET expression. Interestingly, FA co-treatment partially restored DNA methylation as evidenced by global DNA methylation and epigenetic gene expression, and also by compensatory effect via one-carbon metabolism. Genome-wide DNA methylation revealed site-specific hypermethylation at key developmental genes, several of which were reversed with FA. Proteomics analysis identified downregulation of developmentally critical proteins, including those linked to key metabolic processes, while FA co-treatment reversed expression of several such proteins. Integrative methylome–proteome analysis revealed the coordinated regulation of target genes that are linked to congenital abnormalities. Together, these findings offer mechanistic insight into ASM-induced teratogenesis and support FA’s potential to mitigate epigenetic and proteomic disruptions. This integrated OMICs based approach identifies key biomarkers which can be used for therapeutic monitoring and help in optimizing maternal epilepsy management.

## 1. Introduction

Antiseizure medications (ASMs) act on multiple molecular targets to reduce neuronal excitability, providing epileptic patients with adequate seizure control [1]. One-third of epileptic individuals receiving ASMs in prenatal clinics are women who are of reproductive age. Pregnant epileptic women who take medications for tonic–clonic seizures bear the risk of serious harm and teratogenic effects on the developing fetus [2]. In India, primary healthcare facilities provide first-generation ASMs such as valproate (VPA), phenytoin (PHT), carbamazepine (CBZ), and phenobarbitone (PB) through public health programs, making them an accessible and cost-effective option for epilepsy management [3,4]. Additionally, medications like PHT and CBZ continue to be the first-choice ASM for treating focal epilepsy [5]. Despite the fact that most epileptic mothers give birth to healthy infants, the likelihood of significant congenital abnormalities in the progeny is increased by two to three times. The teratogenic effects of ASMs are the primary cause of this increase [6]. The major malformations that arise during organogenesis are structural abnormalities, which can result in dysfunction or even premature death. The most prevalent congenital birth defects include neural tube defects, congenital heart disease, orofacial clefts, and urogenital defects [7]. According to the study conducted by the Global Burden of Disease in 2019, congenital birth defects were among the top ten causes of death for children under the age of five [8].

Building on this, in 1972, Speidel and Meadow observed that individuals undergoing treatment with ASMs experienced a reduction in serum FA levels, which consequently heightened the risk of malformations [9]. Clinical studies suggest that FA supplementation can substantially decrease the occurrence of birth defects in children born to mothers undergoing treatment with ASMs. Therefore, it is recommended that women who are considering pregnancy and those in the early months of gestation take a daily dose of 5 mg of folic acid [10]. Extensive research over the decades has identified folate deficiency as a risk factor for neural tube defects (NTDs) [11]; however, disruptions in overall methylation metabolism have also been associated with NTD development [12]. Furthermore, while ASMs pose teratogenic risks during pregnancy, most are not contraindicated during breastfeeding, as studies report low drug transfer into breast milk and no significant adverse neurodevelopmental outcomes in exposed infants, though individualized risk assessment remains essential [13].

Considering these birth defects, the developmental origins of health and disease (DOHaD) hypothesis posits that the early-life environment plays a critical role in determining health outcomes over the course of a lifetime [14]. In this context, epigenetic changes, particularly DNA methylation, are regarded as significant mechanisms through which environmental influences can affect health in later stages of life [15]. DNA methylation at cytosine positions in CpG dinucleotides is a key factor in regulating gene expression and maintaining genomic stability, and is essential for various developmental processes [16]. During embryogenesis, the epigenomic landscape is highly vulnerable to environmental factors, as this is the crucial period when the DNA methylation patterns necessary for proper tissue development are established [17]. The fact that the human epigenome is susceptible to environmental signals, not only during development, but also throughout life, is leading to the realization that these pharmacological drugs have the potential to modify gene expression with long-lasting effects [18].

Further supporting this epigenetic perspective, in the realm of one-carbon metabolism, is folate, which is an important factor required for the production of S-adenosylmethionine, the primary methyl donor that supports the methylation of DNA [19]. Thus, the presence of folate significantly affects the development and sustainability of DNA methylation patterns. Studies have shown that maternal folate levels throughout pregnancy are associated with changes in the DNA methylation profiles of offspring, with implications evident in both the short and long term [20].

Clinical investigations have revealed the adverse clinical outcome of CBZ, PHT, and VPA on the fetus during pregnancy. Valproic acid (VPA) is a well-known epigenetic modulator that alters DNA’s chemical structure without changing its ability for base-paring [21]. However, there remains insufficient evidence regarding the mechanisms by which these medications cause malformations and teratogenic effects on fetal embryonic cells. In proposing a fundamental hypothesis concerning the safety of administering ASMs during pregnancy and their potential epigenetic toxicity, especially in terms of implications for future generations, our goal was to explore whether ASMs can influence the human epigenome using an integrated analysis of epigenomic and proteomic analysis at genome-wide and proteome-wide screening and relate them to key malformations induced by ASMs.

## 2. Results

### 2.1. Antiseizure Medications Induce Global DNA Hypomethylation Reversed by Folic Acid Co-Treatment

**Figure 1:**
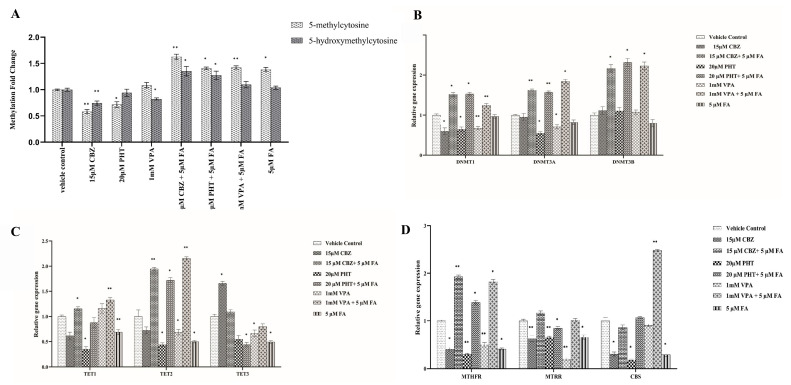
Effects of antiseizure drugs and folic acid on global DNA methylation and the transcriptional regulation of epigenetic and one-carbon metabolism genes. (**A**) In vitro evaluation of global DNA methylation levels (5-methylcytosine and 5-hydroxymethylcytosine) following treatment with antiseizure drugs alone and in combination with folic acid. (**B**) Relative gene expression levels of DNA methyltransferase genes (DNMT1, DNMT3A, and DNMT3B). (**C**) Relative gene expression levels of DNA demethylase genes (TET1, TET2, and TET3). (**D**) Gene expression analysis of one-carbon metabolism-related genes (MTHFR, MTRR, CBS) under different treatment conditions. **Abbreviations**: CBZ—carbamazepine, PHT—phenytoin, VPA—valproic acid, FA—folic acid, DNMT1—DNA methyltransferases 1, DNMT3A—DNA methyltransferases 3 Alpha, DNMT3B—DNA methyltransferases 3 Beta, TET1—Ten-Eleven Translocase 1, TET2—Ten-Eleven Translocase 2, TET3—Ten-Eleven Translocase 3, MTHFR—Methylenetetrahydrofolate Reductase, MTRR—Methionine Synthase Reductase, CBS—Cystathionine Beta-Synthase. * denotes significance—*p*-value < 0.05 flagged with one star (*), *p*-value < 0.01 (**).

The administration of 15 μM carbamazepine (CBZ), 20 μM phenytoin (PHT), and 1 mM valproic acid (VPA) for 24 h led to a significant reduction in global levels of both 5-methylcytosine (5-mC) and 5-hydroxy-methyl-cytosine (5-hmC) compared to the untreated vehicle control group (Figure 1A). Specifically, 5-mC levels were significantly reduced by 0.58-fold following 15 μM CBZ treatment (*p* = 0.004) and by 0.71-fold following 20 μM PHT treatment (*p* = 0.045). Similarly, 5-hmC levels decreased by 0.74-fold in response to 15 μM CBZ (*p* = 0.006) and by 0.82-fold with 1 mM VPA (*p* = 0.04).

In contrast, co-treatment with 5 μM FA resulted in a notable increase in both 5-mC and 5-hmC levels. Specifically, combined treatment with 15 μM CBZ + 5 μM FA, 20 μM PHT + 5 μM FA, and 1 mM VPA + 5 μM FA resulted in 1.62-fold (*p* = 0.003), 1.4-fold (*p* = 0.043), and 1.42-fold (*p* = 0.002) increases in 5-mC levels, respectively. Correspondingly, 5-hmC levels were elevated by 1.3-fold (*p* = 0.035), 1.2-fold (*p* = 0.021), and 1.09-fold in these respective treatments. These data collectively demonstrate that ASMs elicit significant global DNA hypomethylation, which is effectively reversed upon FA supplementation, highlighting the epigenetic modulatory potential of FA against ASM-induced methylation perturbations (Figure 1A).

### 2.2. ASM-Induced Transcriptomic Alterations in Core Epigenetic Regulators and Their Modulation by Folic Acid

To elucidate whether ASM-induced changes in global DNA methylation are linked to the altered transcription of key epigenetic modifiers, we quantified the mRNA expression of DNA methyltransferases (DNMT1, DNMT3A, DNMT3B) and ten-eleven translocation enzymes (TET1, TET2, TET3) in HEK293 cells after 24 h exposure to 15 μM CBZ, 20 μM PHT, or 1 mM VPA (Figure 1B,C; Supplementary File S1).

DNMT1 expression was significantly reduced by 0.6-fold (*p* = 0.017), 0.64-fold (*p* = 0.014), and 0.67-fold (*p* = 0.0071) with CBZ, PHT, and VPA, respectively. DNMT3A was downregulated by 0.54-fold (*p* = 0.031) with PHT and 0.7-fold (*p* = 0.0107) with VPA, while DNMT3B remained unchanged. TET1 decreased by 0.34-fold (*p* = 0.028) with PHT, while TET2 decreased by 0.43-fold (*p* = 0.029) and 0.68-fold (*p* = 0.036) with PHT and VPA, respectively. TET3 showed a significant increase (1.66-fold, *p* = 0.0104) with CBZ but was reduced by 0.66-fold (*p* = 0.0454) with VPA.

FA co-administration markedly reversed several ASM-induced transcriptional changes. CBZ + FA significantly upregulated DNMT1 (1.51-fold, *p* = 0.0129), DNMT3A (1.62-fold, *p* = 0.009), DNMT3B (2.16-fold, *p* = 0.0191), TET1 (1.15-fold, *p* = 0.0151), and TET2 (1.94-fold, *p* = 0.006). PHT + FA elevated DNMT1 (1.53-fold, *p* = 0.012), DNMT3A (1.57-fold, *p* = 0.0084), DNMT3B (3.31-fold, *p* = 0.013), and TET2 (1.721-fold, *p* = 0.013), while TET3 was downregulated (0.44-fold, *p* = 0.0416). VPA + FA significantly increased DNMT1 (1.24-fold, *p* = 0.0045), DNMT3A (1.84-fold, *p* = 0.0121), DNMT3B (3.23-fold, *p* = 0.013), TET1 (1.32-fold, *p* = 0.0085), and TET2 (2.15-fold, *p* = 0.0068). These data demonstrate ASM-induced disruption of epigenetic gene networks, which may underlie global methylation changes and highlight folic acid’s potential to transcriptionally re-equilibrate key methylation-associated enzymes.

### 2.3. ASMs Disrupt One-Carbon Metabolic Gene Networks: Restorative Potential of Folic Acid

To evaluate whether global methylation changes were linked to alterations in one-carbon metabolism, we assessed the expression of key genes—MTHFR, MTRR, and CBS—in HEK293 cells following 24 h treatment with ASMs (15 μM CBZ, 20 μM PHT, and 1 mM VPA) (Figure 1D; Supplementary File S2). ASM exposure significantly downregulated MTHFR by 0.4-fold (*p* = 0.045), 0.3-fold (*p* = 0.002), and 0.5-fold (*p* = 0.0034) for CBZ, PHT, and VPA, respectively. MTRR expression decreased by 0.62-fold (*p* = 0.006), 0.6-fold (*p* = 0.003), and 0.2-fold (*p* = 0.0035), respectively, while CBS was reduced by 0.3-fold (*p* = 0.049) and 0.18-fold (*p* = 0.042) under CBZ and PHT, respectively.

FA co-treatment mitigated these effects. MTHFR expression increased by 1.92-fold (*p* = 0.0091), 1.38-fold (*p* = 0.012), and 1.82-fold (*p* = 0.026) with CBZ + FA, PHT + FA, and VPA + FA, respectively. CBS was significantly upregulated (2.4-fold, *p* = 0.0017) with VPA + FA, while MTRR was downregulated (0.84-fold, *p* = 0.017) with PHT + FA.

### 2.4. Deciphering the Dynamics of Differential DNA Methylation

The impact of global methylation patterns and the expression patterns of the genes involved in methylation homeostasis was further investigated using genome-wide methylation screening using Infinium 935K EPIC v2 BeadChip array upon ASM monotherapy and followed by FA supplementation.

#### Folic Acid Mitigates ASM-Induced Hypermethylation

**Figure 2:**
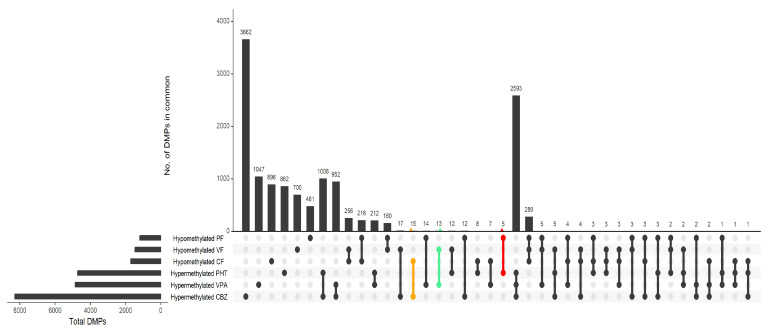
Upset plot visualizes the overlap and distinctness of differentially methylated CpG sites (DMPs) across antiseizure drug treatments, with and without folic acid (FA) supplementation. Vertical bars represent DMPs shared between comparisons, while horizontal bars show the total number of DMPs per treatment. Highlighted regions indicate overlaps: red for hypermethylated PHT (phenytoin) and hypomethylated PF (phenytoin + FA); green for hypermethylated VPA (valproic acid) and hypomethylated VF (valproic acid + FA); and yellow for hypermethylated CBZ (carbamazepine) and hypomethylated CF (carbamazepine + FA), black dots represent unique DMPs to that treatment, while connected dots indicate DMPs shared across multiple treatment conditions.

A substantial proportion of differentially methylated CpG sites (DMPs) demonstrated pervasive hypermethylation across all ASM regimens, with 2593 CpG sites consistently hypermethylated across treatments with CBZ, PHT, and VPA. Furthermore, the study revealed that 3662 CpGs were hypermethylated exclusively in the CBZ treatment, while 1047 CpGs were uniquely hypermethylated in the VPA treatment, and 862 CpGs were specifically hypermethylated in the PHT treatment (Supplementary Files S3–S9). These results suggest distinct epigenetic signatures specific to each drug, as depicted in the Upset plot (Figure 2). The incorporation of FA notably attenuated this hypermethylation, shifting a subset of DMPs toward hypomethylation in the combination treatments—phenytoin + folic acid (PF), valproic acid + folic acid (VF), and carbamazepine + folic acid (CF). In the comparison between the hypermethylated CBZ group and the hypomethylated CF group, 15 probes were identified, with 7 located in the promoter region. These probes, which overlap between the two groups, included cg15825819, cg10724635, cg04650055, cg27191207, cg00061551, cg24013293, and cg10468550, highlighting critical regulatory sites affected by CBZ treatment. The hypermethylated PHT group showed significant concordance with the hypomethylated PF group, encompassing five probes, with one situated within the promoter region (cg21106446) reflecting key regions where FA supplementation may mitigate the hypermethylation effects induced by PHT. Additionally, the association between hypermethylated VPA and hypomethylated VF uncovered 13 probes, with 2 residing in the promoter region: cg16784828 and cg09472506, further underscoring the potential modulatory role of FA on VPA-induced hypermethylation at these critical regulatory loci. Collectively, these findings illuminate the intricate epigenetic landscape shaped by ASM exposure and its modulation by FA, particularly at promoter regions pivotal for gene expression regulation.

**Figure 3:**
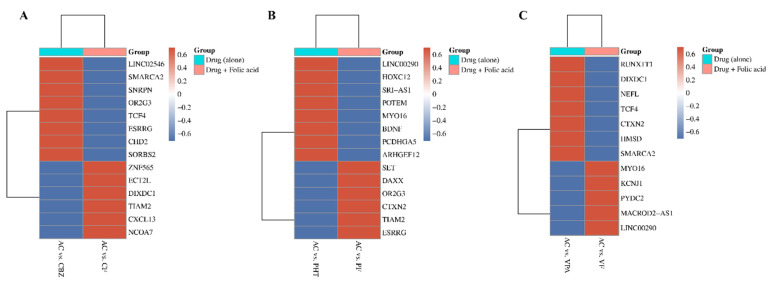
Heatmap illustrating the reversal of antiseizure drug-induced DNA methylation changes by folic acid supplementation. Panels represent methylation patterns for (**A**) carbamazepine, (**B**) phenytoin, and (**C**) valproic acid treatments. The color gradient reflects Delta beta (Δβ) values, with red indicating hypermethylation (Δβ > 0.2) and blue indicating hypomethylation (Δβ < 0.2). The gene sets on the *y*-axis correspond to top 10 with significant methylation (FDR adjusted *p*-value < 0.05) changes across different treatment conditions.

The heatmap (Figure 3) illustrates how FA supplementation reverses ASM-induced DNA methylation changes, quantified by Delta beta (Δβ) values. Delta beta measures methylation differences, with red indicating hypermethylation (Δβ > 0.2) and blue indicating hypomethylation (Δβ < −0.2). Genes with significant changes in methylation status across CBZ, PHT, and VPA treatments are displayed, focusing on the top 10 with FDR-adjusted *p*-values < 0.05. Each panel compares methylation patterns between two groups: ASM alone and ASM combined with FA, highlighting FA’s potential role in mitigating ASM-induced DNA methylation. The clustering along the *y*-axis highlights genes that exhibit similar methylation responses across treatment conditions, with notable overlap in key genes such as *LINC00290*, *MYO16*, *CTXN2*, *OR2G3*, *ESRRG*, *TIAM2*, *SMARCA2*, *TCF4*, and *DIXDC1*.

### 2.5. Proteomic Profiling Reveals Distinct and Overlapping Signatures of Antiseizure Medications and Folic Acid Co-Therapy

**Figure 4:**
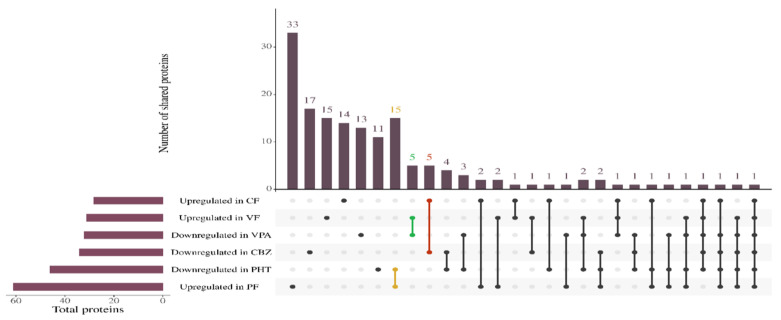
The Upset plot visualizes the overlap and distinctness of differentially expressed proteins across treatment conditions. Vertical bars represent proteins shared between comparisons, while horizontal bars show the total number of proteins per treatment. Highlighted regions indicate overlaps: red for downregulated proteins in CBZ (carbamazepine) and upregulated in CF (carbamazepine + FA); green for downregulated proteins in VPA (valproic acid) and upregulated in VF (valproic acid + FA); and yellow for downregulated proteins in PHT (phenytoin) and upregulated in PF (phenytoin + FA), black dots represent unique DMPs to that treatment, while connected dots indicate DMPs shared across multiple treatment conditions.

To delineate the proteomic landscape associated with ASM exposure and to evaluate the modulatory role of FA, a label-free quantitative proteomic analysis was conducted using LC-MS/MS. High-confidence protein identification was stringently filtered using a 1% false discovery rate (FDR) and the presence of a minimum of two unique peptides (Supplementary File S10). Based on this stringency, under ASM monotherapy, 93, 101, and 147 proteins were identified in CBZ-, PHT-, and VPA-treated groups, respectively. Co-administration of FA markedly enhanced protein identifications, with 211 proteins in the CBZ + FA (CF) group, 107 in PHT + FA (PF), and 150 in VPA + FA (VF). Differential expression was assessed by computing abundance ratios relative to control; proteins with abundance ratios >1 was designated as downregulated, while those <1 were considered upregulated. A statistical threshold of *p* < 0.05 was applied for significance (Supplementary Files S11–S20). To visualize the extent of FA-mediated proteomic modulation, an Upset plot (Figure 4) was employed to capture the intersection of differentially expressed proteins across treatment conditions. Importantly, distinct overlaps were observed between proteins downregulated under ASM monotherapy and those upregulated in the corresponding FA co-treatment groups.

**Figure 5:**
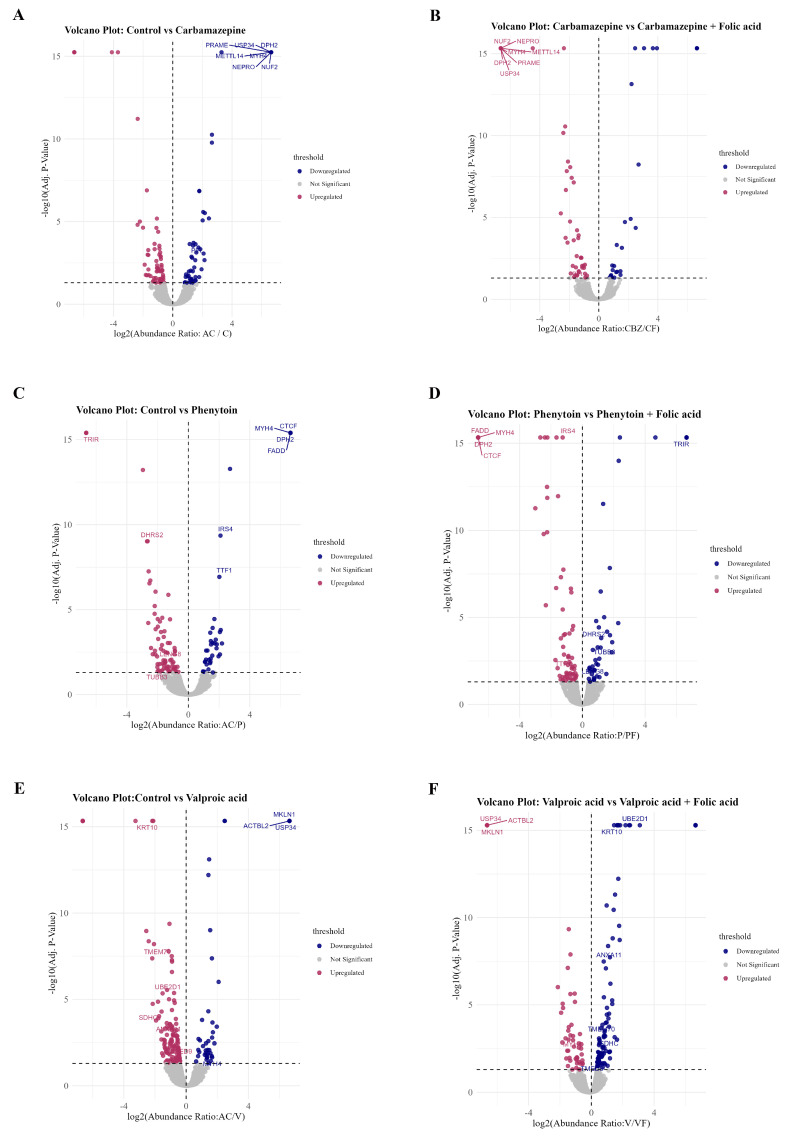
Volcano plots illustrating protein-level alterations induced by ASMs and their reversal upon FA co-treatment. Plots display differential protein expression (log_2_ fold change, *x*-axis) against statistical significance (−log_10_ FDR-value, *y*-axis) for each comparison: (**A**,**B**) CBZ vs. CF; (**C**,**D**) PHT vs. PF; and (**E**,**F**) VPA vs. VF. Proteins significantly dysregulated (*p* < 0.05) are highlighted.

Specifically, 15 proteins were commonly downregulated in PHT and upregulated in PF, 5 proteins overlapped between VPA and VF, and 5 overlapped between CBZ and CF. These conserved overlaps point to a core set of FA-responsive proteins that may counteract ASM-induced suppression. This compensatory effect was corroborated by volcano plot analyses (Figure 5A–F), which illustrate treatment-specific protein alterations across each ASM-FA comparison: control vs. CBZ and CBZ vs. CF (Figure 5A,B), control vs. PHT and PHT vs. PF (Figure 5C,D), and control vs. VPA and VPA vs. VF (Figure 5E,F). Notably, several proteins such as FADD, TRIM37, CTCF, and KRT10 exhibited significant downregulation under ASM monotherapy, but demonstrated upregulation upon FA co-treatment. These findings support a mechanistic role for FA in ameliorating ASM-induced proteomic perturbations and underscore its therapeutic potential in preserving cellular protein homeostasis during ASM exposure.

### 2.6. Gene Ontology Analysis of Differential Methylome and Proteome After ASM Treatment

#### 2.6.1. Gene Ontology of Methylome Identifies Enrichment in Developmental Process

**Figure 6:**
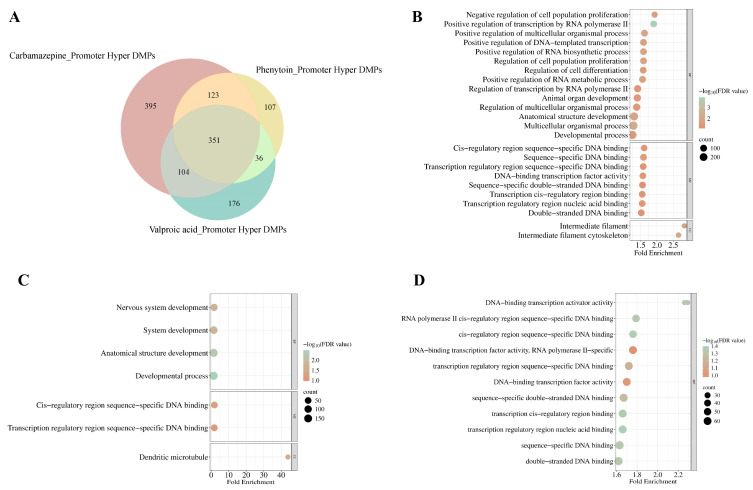
Promoter hypermethylated probes and associated gene ontology enrichment across ASM treatments. (**A**) Venn diagram illustrating the number of significantly hypermethylated probes identified in response to CBZ, PHT, and VPA treatments. (**B**–**D**) Gene ontology (GO) enrichment analysis of DMPs for carbamazepine (**B**), phenytoin (**C**), and valproic acid (**D**). The *x*-axis represents fold enrichment and the *y*-axis lists enriched GO terms from biological process (BP), cellular component (CC), and molecular function (MF) categories. Bubble color indicates −log10 (FDR value) and bubble size reflects the number of genes associated with each GO term.

To delineate promoter-specific methylation alterations induced by ASMs, we performed a Venn analysis of DMPs exhibiting significant promoter (encompassing TSS1500 and TSS200) hypermethylation (FDR adjusted *p*-value < 0.05) in response to CBZ, PHT, and VPA treatments (Figure 6A). CBZ exhibited the highest number of uniquely hypermethylated probes (*n* = 395), followed by VPA (*n* = 176) and PHT (*n* = 107), with 351 DMPs commonly hypermethylated across all three conditions. The gene ontology (GO) enrichment analysis of these DMPs identified distinct patterns of biological and cellular processes influenced by each drug (Supplementary Files S21–S23).

In the CBZ-treated group, significant enrichment is observed in biological processes pertinent to developmental mechanisms (FDR = 2.65 × 10^−2^), anatomical structure morphogenesis (FDR = 1.08 × 10^−2^), and the regulation of cell proliferation (FDR = 1.21 × 10^−2^) and differentiation (FDR = 2.51 × 10^−2^). Further analysis of cellular components revealed a notable association with the intermediate filament cytoskeleton (FDR = 9.17 × 10^−3^), suggesting a critical role in preserving cytoskeletal integrity and its implications for cellular dynamics (Figure 6B, Supplementary File S21).

For the PHT-treated group, enriched terms were predominantly linked to nervous system development (FDR = 1.72 × 10^−2^), alongside general developmental processes (FDR = 3.24 × 10^−3^) and structural development of anatomical components (FDR = 5.26 × 10^−3^), with cellular component analysis pinpointing enrichment within dendritic microtubules (FDR = 2.35 × 10^−2^), underscoring the drug’s impact on dendritic structure and neural network organization (Figure 6C, Supplementary File S22).

Conversely, for the VPA-treated group, no significant enrichment was detected within biological processes; however, molecular function analysis revealed associations with DNA-binding transcription activity (FDR = 4.83 × 10^−2^), including specific enrichments for double-stranded DNA binding (FDR = 4.60 × 10^−2^) and sequence-specific DNA interactions (FDR = 4.61 × 10^−2^). These observations align with the modulation of transcriptional regulation as a key mechanism influenced by VPA-induced epigenetic alterations (Figure 6D, Supplementary File S23). Collectively, these findings emphasize the differential regulatory landscapes shaped by ASM treatments, highlighting their distinct epigenetic imprints on gene expression and cellular functionality.

#### 2.6.2. Gene Ontology of Proteome Identifies Regulation of Mitochondrial and Nuclear Pathways in Response to Antiseizure Medications

**Figure 7:**
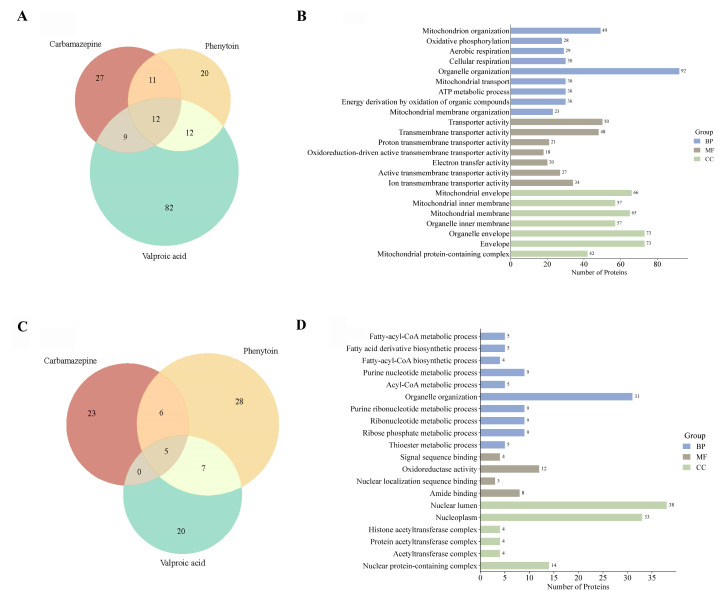
GO enrichment analysis of differentially expressed proteins in response to ASMs. Venn diagrams illustrate the overlap of significantly upregulated (**A**) and downregulated (**C**) proteins (adjusted *p* < 0.05) among CBZ, PHT, and VPA treatments. Corresponding GO enrichment analysis of these protein subsets is shown in (**B**,**D**), respectively, categorized into biological processes (BP), molecular functions (MF), and cellular components (CC).

To elucidate the functional impact of ASM exposure on the cellular proteome, we performed comparative GO enrichment analyses of differentially expressed proteins across CBZ, PHT, and VPA treatments. Venn diagram analysis of upregulated proteins (Figure 7A) revealed that VPA uniquely induced the highest number of proteins (*n* = 82), with 12 proteins commonly upregulated across all treatment groups. GO enrichment of these proteins (Figure 7B) demonstrated a marked overrepresentation of mitochondrial-related processes, including “mitochondrion organization,” “oxidative phosphorylation,” and “aerobic respiration,” underscoring a concerted upregulation of mitochondrial bioenergetics and structural remodeling. Conversely, downregulated proteins (Figure 7C) showed more limited overlap, with 20 proteins uniquely suppressed by VPA and only 5 shared across all treatments. Functional annotation of these downregulated proteins (Figure 7D) revealed significant enrichment for biological processes associated with nucleotide metabolism, ribonucleoprotein biogenesis, and nuclear-localized acetyltransferase complexes. Collectively, these results highlight the distinct proteomic signatures elicited by each ASM, with VPA exerting the most extensive effects on both mitochondrial and nuclear regulatory networks (refer Supplementary Files S24 and S25 for more details).

#### 2.6.3. ASM–Induced Proteomic Alterations to Developmental Disorders

**Figure 8:**
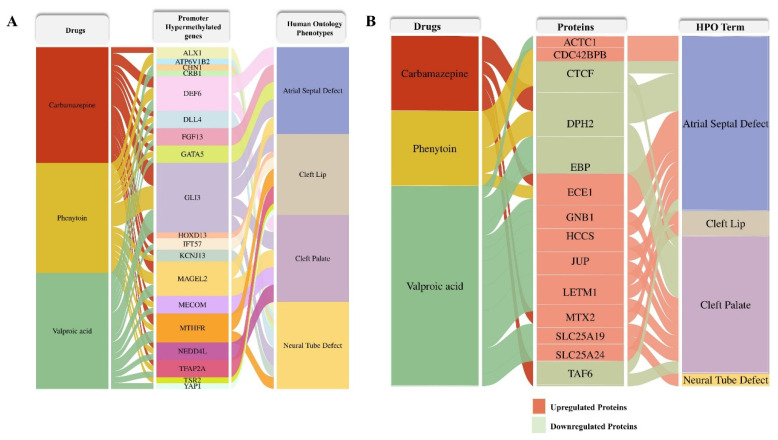
Alluvial plot illustrating the association between ASM treatments and their corresponding developmental phenotypes derived from the Human Phenotype Ontology (HPO) database associated with differentially methylated genes (**A**) and dysregulated proteins (**B**). The left panel lists the ASMs (carbamazepine, phenytoin, valproic acid), the middle panel shows drug-responsive proteins, and the right panel presents HPO terms associated with those proteins, including atrial septal defect, cleft lip, cleft palate, and neural tube defect. Protein regulation is color-coded: upregulated (salmon) and downregulated (light green).

To elucidate the potential phenotypic consequences of ASM exposure, we constructed an alluvial plot linking differentially methylated genes and differentially expressed proteins identified in response to carbamazepine, phenytoin, and valproic acid treatments with their associated human phenotypes. Human Phenotype Ontology (HPO) terms corresponding to each differentially methylated gene (Figure 8A) and dysregulated protein (Figure 8B) were systematically retrieved from the HPO database (http://www.human-phenotype-ontology.org, accessed on 13 June 2025). Several proteins exhibited associations with congenital anomalies, including atrial septal defect, cleft lip, cleft palate, and neural tube defect, highlighting the potential teratogenic pathways perturbed by these drugs.

## 3. Discussion

This study comprehensively investigates the molecular perturbations induced by first-generation antiseizure medications (ASMs), integrating high-resolution epigenomic and proteomic analyses to delineate their impact on developmental programming. By coupling genome-wide DNA methylation profiling (Supplementary Files S3–S9) with quantitative LC-MS/MS-based proteomics (Supplementary Files S11–S20), we reveal the coordinated dysregulation of epigenetic regulators, one-carbon metabolism genes, and developmental proteins—many of which are partially restored by folic acid co-treatment. HEK293 cells, derived from human embryonic kidney, offer both experimental reproducibility and developmental relevance, making them an ideal model for investigating ASM-induced epigenetic changes. Their suitability is supported by prior studies [22], including our confirmation of VPA’s HDAC inhibitory activity (unpublished data; available upon request for peer review) and the literature linking prenatal ASM exposure to congenital anomalies such as multicystic dysplastic kidney (MCDK) [23], highlighting disrupted nephrogenesis as a key developmental concern. It is well established that maternal exposure to different agents trigger epigenetic mechanisms, altering the gene expression and, consequently, impairing embryo development [24]. DNA methylation is a critical epigenetic mechanism regulating gene expression. To assess whether first-generation ASMs influence this process, we evaluated global methylation levels using 5-methylcytosine (5-mC) and 5-hydroxymethylcytosine (5-hmC) as proxies. All three ASMs—CBZ, PHT, and VPA—significantly reduced global 5-mC levels, while CBZ and VPA also elevated 5-hmC, indicating active demethylation. To investigate underlying mechanisms, we examined key methylation regulators and found a marked downregulation of DNMT1 and DNMT3A, as well as the demethylation enzymes TET1, TET2, and TET3, following ASM exposure. These findings are consistent with earlier reports implicating VPA as a DNMT inhibitor [22], and notably, we observed a pronounced suppression of DNMT1 and DNMT3A, accompanied by an increase in TET1 expression, highlighting the dual modulation of methylation machinery by ASMs, particularly VPA [25]. Methylation concordance has been closely linked to the spatial co-localization of DNMT and TET enzymes and has been shown to correlate strongly with gene expression regulation [26]. Therefore, the coordinated alterations in DNMT and TET expression observed in this study likely influence DNA methylation dynamics and downstream gene expression. Collectively, these findings suggest that ASMs modulate key epigenetic regulators, contributing to the observed reduction in global DNA methylation levels.

Furthermore, clinical studies suggest that being exposed to first generation ASMs during pregnancy can cause several malformations, including cleft palate, neural tube defects, hypospadias, and cardiovascular defects [7]. Despite known teratogenic risks, particularly with VPA, first-generation ASMs continue to be widely used in India due to their affordability and clinical effectiveness, especially in low-resource settings; data from the Kerala Registry of Epilepsy and Pregnancy (KREP) also indicate that while VPA poses a higher malformation risk, older ASMs remain commonly prescribed, with newer ASMs associated with a higher incidence of generalized tonic–clonic seizures during pregnancy [27]. Studies have suggested that the use of FA at doses ranging from 4 to 5 mg daily during pregnancy may lead to a reduction in teratogenic effects by approximately 50% [10]. The developmental toxicity of ASMs is influenced by multiple factors, including disruptions in folate metabolism. To address this, we employed FA supplementation alongside ASMs, selecting optimal dosages based on prior dose–response analyses. A population-based study by the National Birth Defects Prevention group reported that higher periconceptional intake of methyl donors was associated with a reduced risk of neural tube defects among women meeting FA recommendations [28]. In our study, ASM treatment alone reduced the expression of key one-carbon metabolism genes (MTHFR and MTRR), whereas co-treatment with FA restored MTRR expression and upregulated MTHFR. Notably, FA supplementation also enhanced the expression of epigenetic regulators (DNMT1, DNMT3A, DNMT3B, TET1, and TET2), suggesting a protective role in maintaining both methylation potential and epigenetic homeostasis during ASM exposure.

Building on our findings of reduced expression of epigenetic regulators following ASM treatment, we explored the potential link between altered DNA methylation and developmental abnormalities. Prenatal ASM exposure has been shown to induce heritable epigenetic modifications, particularly affecting DNMT1—a key enzyme in maintaining DNA methylation during replication [29], whose dysfunction is embryonically lethal [30]. Aberrant methylation patterns, whether hypo- or hypermethylation, can disrupt gene regulation, compromise genomic stability [31], and contribute to developmental defects such as spina bifida and other congenital anomalies [32].

Our genome-wide methylome analysis revealed a notable epigenetic paradox; although ASM exposure led to a global reduction in DNA methylation levels, site-specific analyses uncovered widespread hypermethylation at distinct CpG loci. This pattern aligns with prior observations that global DNA hypomethylation can coexist with localized hypermethylation at gene regulatory regions [33], suggesting that ASMs may selectively target gene promoters to modulate transcriptional activity in a context-dependent manner. FA co-administration further amplified hypermethylation, particularly in the CBZ + FA and PHT + FA groups relative to VPA + FA, reflecting distinct drug-specific epigenetic responses to FA supplementation. Notably, consistent hypermethylation at cg06914070 within the TET2 promoter across all ASM and ASM + FA conditions suggests interference with DNA demethylation machinery by modulating TET2 expression [34,35]. Similarly, the MTHFR gene exhibited hypermethylation at cg11544334 and cg22059149, which corroborates earlier evidence that ASMs impair one-carbon metabolism and influence folate-dependent methylation processes [36]. FA supplementation intensified these effects, especially under CBZ treatment. Furthermore, pronounced differential methylation in intergenic regions suggests that ASMs elicit widespread epigenetic reprogramming beyond promoter regions, potentially driving transcriptional repression of essential genes [37].

Folic acid, as a methyl donor, modulates DNA methylation and counteracts ASM-induced epigenetic disruptions. In our study, FA co-treatment reversed aberrant methylation patterns at key regulatory loci, supporting its role in preserving developmental gene expression and mitigating ASM-associated teratogenic risks [38]. Gene ontology analysis revealed that ASM treatments distinctly impact developmental and cellular pathways, underscoring their unique epigenetic signatures. CBZ treatment enriched processes related to morphogenesis and cell proliferation, suggesting disruption of key developmental programs [39]. PHT selectively affected nervous system development, particularly dendritic microtubule organization, aligning with its known impact on neuronal architecture [40]. In contrast, VPA treatment enriched transcription-related functions, consistent with its epigenetic modulation of gene expression via DNA-binding transcription factors [41].

This study reveals dynamic, gene-specific methylation shifts in key developmental regulators under ASM exposure, with partial reversal upon FA supplementation. Notably, *MYO16*, essential for synaptic structure [42], exhibited promoter hypermethylation (cg04250084) across all ASMs, while FA co-treatment induced hypomethylation at cg00757772. Similarly, SETBP1, a chromatin regulator [43], showed exon 4 hypermethylation (cg24776880) under ASM-alone treatment, which shifted to hypomethylation at TSS200 (cg05939871) with FA. These findings highlight the context-dependent, ASM- and FA-driven epigenetic remodeling of developmental genes.

Our LC-MS/MS–based proteomics analysis reinforces the mitigating role of FA on ASM-induced proteomic disruptions. Several proteins downregulated under ASM monotherapy were upregulated with FA co-treatment, indicating a potential compensatory effect. For example, NEPRO, a nucleolar protein essential for the preimplantation development and Notch pathway–mediated maintenance of neural progenitors [44], was downregulated in CBZ-treated samples but restored upon FA co-administration. MYH4, a myosin isoform critical for muscle function and commonly affected in ASM-induced myotoxicity, was downregulated across all ASM treatments and reverted upon FA addition, aligning with established reports of ASM-driven muscular toxicity [45]. UBE2D1, previously shown to be upregulated by VPA in SH-SY5Y neuronal cells and associated with hepatocellular carcinoma progression [46], was similarly elevated in VPA monotherapy and reversed with FA, suggesting FA may counteract such adverse proliferative effects. GO enrichment analysis revealed ASM-associated upregulation of mitochondrial pathways, including oxidative phosphorylation, consistent with prior findings of VPA-driven mitochondrial biogenesis [47]. Importantly, integrated methylome and proteome data identified RPL28 as a candidate showing concordant epigenetic and proteomic alterations: it exhibited promoter hypermethylation and concurrent protein downregulation, implicating a potential mechanism of transcriptional repression via DNA methylation. Collectively, these findings emphasize the epigenetic and proteomic convergence in ASM responses and highlight FA’s capacity to normalize molecular disruptions that may underlie teratogenic risks associated with ASM therapy.

## 4. Materials and Methods

### 4.1. Cell Culturing

Human embryonic kidney cell line HEK293 (ATCC (^®^CRL-1573 ™)) was maintained in Dulbecco’s modification of Eagle’s medium (DMEM; Gibco, Waltham, MA, USA) supplemented with 10% fetal bovine serum (Gibco, Waltham, MA, USA) and 1× antibiotic antimycotic solution (Invitrogen, Carlsbad, CA, USA) in a 37 °C humidified incubator with 5% CO_2_. Short Tandem Repeats (STR) profiling of HEK293 was carried out using the Applied Biosystems AmpFLSTR Identifiler amplification kit. Cell line authentication verified the cell line’s authenticity in accordance with the ATCC (American Tissue Culture Collection) specifications. Cells were plated at a density of 3 × 10^5^ cells per well in 6-well plates, with each plate dedicated to a specific treatment concentration and timing. All the experimental protocols were carried out in triplicates.

### 4.2. Antiseizure Drug Treatment

Carbamazepine (CBZ), phenytoin (PHT), and valproic acid (VPA) were chosen for this study. These drugs were procured from sigma chemical company (Sigma, Aldrich, MO, USA) and were dissolved in dimethyl sulfoxide (DMSO). For control experiments, fresh medium (DMEM as control and DMSO as vehicle control) was added. The final concentration of DMSO in the medium was less than 0.20%. The suitable concentration of ASMs for treatment was determined by MTT (3-(4,5-dimethylthiazol-2-yl)-2,5-diphenyltetrazolium bromide)-based cell viability assay. Cell viability was observed to be 80% up to concentrations of ≤20 µM for CBZ and PHT and ≤1 mM for VPA, which were utilized for subsequent investigations (Supplementary File S26).

### 4.3. Preparation of Folic Acid Solution

Folic acid (FA) was obtained from sigma chemical company (Sigma, Aldrich, St. Louis, MO, USA). FA was diluted in DMSO in 1 µM, 5 µM, and 10 µM concentrations. Following the MTT-based cell viability assay, we chose the FA solution with a 5 µM concentration, which was below the toxic dosage for further experiments. The study design incorporated combination treatments of ASMs with FA, utilizing monotherapy concentrations that closely correspond to therapeutic plasma levels observed in humans [48] (Supplementary File S27). A 24 h treatment period was chosen based on reported Tmax values for these drugs, ensuring that the in vitro exposure duration reflects clinically relevant pharmacokinetic conditions [48] (Supplementary File S27). This approach enhances the translational relevance of the experimental outcomes. Initially, cultured cell lines were subjected to monotherapy with ASMs at concentrations of 15 μM for CBZ, 20 μM for PHT, and 1 mM for VPA administered for 24 h. Following this, combination treatments were introduced, comprising 15 μM CBZ with 5 μM FA, 20 μM PHT with 5 μM FA, and 1 mM VPA with 5 μM FA, each administered for 24 h.

### 4.4. DNA Extraction from Cell Line and Quantification of Global DNA Methylation

DNA was extracted from cell lines (treated and untreated) using Pure Link ^®^ Genomic DNA Mini kit (Invitrogen, Carlsbad, CA, USA; Cat. No. K1820-01), according to the manufacturer’s instructions. DNA purity was assessed spectrophotometrically using the Nanodrop ND-1000 instrument (Thermo Scientific, Waltham, MA, USA). DNA integrity of the samples was checked by gel electrophoresis. Quantification of global DNA methylation (5-mC) and hydroxymethylation (5-hmC) were performed using colorimetric assay, a MethylFlash™ Methylated DNA 5-mC Quantification kit (Cat No. P-1034) and a MethylFlash™ hydroxy methylated DNA 5-hmC Quantification kit (Cat No. P-1036), respectively (Epigentek, Farmingdale, NY, USA), as per manufacturer’s instructions.

### 4.5. RNA Extraction and Real-Time Quantitative PCR Analysis

Total RNA was extracted and purified from the cell lines using the Trizol reagent (Invitrogen, Carlsbad, CA, USA; 15596026), according to the manufacturer’s instructions. The concentration of total RNA was assessed spectrophotometrically using the Nanodrop ND-1000 instrument (Thermo Scientific). One microgram of total RNA was used for cDNA synthesis using a PrimeScript™ RT reagent kit (Takara, Osaka, Japan; RR037A) as per the manufacturer’s instructions. RT-PCR was performed using the TB Green^®^ Premix Ex Taq™ II (Takara, Osaka, Japan). MRNA expression levels of epigenetic genes (*DNMT1, DNMT3A, DNMT3B, TET1, TET2* and *TET3*) and one-carbon metabolism pathway genes (*MTHFR, MTRR* and *CBS*) were evaluated using real-time PCR with either TaqMan gene expression assay or SYBR Green-based expression assay (Takara, Osaka, Japan). SYBR Green primers were designed using QuantPrime and TaqMan probes were purchased from Applied Biosystems. The details of the primers are given in Supplementary File S28. The real-time PCR data were captured using QuantStudio™ 5 Real-time PCR (Applied Biosystems, Foster City, CA, USA). Data were analyzed using the comparative ∆∆Cq method [49]. β-actin (*ACTB*) was used as the internal control for each sample. We have compared the effects of ASMs using DMSO as a vehicle control. Real-time PCR was conducted in accordance with the Minimum Information for Publication of Quantitative Real-Time PCR Experiment guidelines [50].

### 4.6. Genome—Wide DNA Methylation Analysis

The Infinium Methylation EPIC v2 BeadChip array, targeting 935,000 CpG sites genome-wide, was used according to the manufacturer’s instructions to quantitatively assess CpG methylation status. Genomic DNA was extracted from HEK293 cells treated with ASM monotherapy (15 μM CBZ, 20 μM PHT and 1 mM VPA) and combination therapy with FA (15 μM CBZ, 20 μM PHT and 1 mM VPA each with 5 μM FA) using the PureLink^®^ Genomic DNA Mini Kit (Invitrogen, Cat. No. K1820-01) following the manufacturer’s protocol. The extracted DNA was subsequently treated with bisulfite using the EZ DNA Methylation™ Kit (Zymo Research, Irvine, CA, USA) in accordance with the manufacturer’s instructions. The bisulfite-converted DNA was amplified and enzymatically fragmented, then applied to the array for hybridization and single-base extension, after which the fluorescently labeled BeadChip was scanned using the Illumina iScan system (Illumina, San Diego, CA, USA). Raw intensity data (*.idat) from the Illumina iScan system were imported into the Genome Studio Methylation module with appropriate sample grouping and error models. Quality control was conducted using the minfi, ChAMP, and RnBeads packages to address technical assay failures, sample mix-ups, and batch effects. Data filtering was performed using the ChAMP pipeline, which included removal of probes with detection *p*-values > 0.01 (indicating unreliable signals), probes with <3 beads in at least 5% of samples, non-CpG probes, SNP-associated probes, multi-hit probes, and those located on sex chromosomes (X and Y). Following all quality control and filtering steps, a total of 102,677 high-quality probes were retained for analysis. The methylation status of each CpG site in samples that passed quality control was quantified as a β-value, which had been normalized to account for color bias, background level, and quantile normalization across arrays. The β-value was calculated as the ratio of the fluorescent signal intensity from the methylated (M) probe to the combined intensity from both methylated (M) and unmethylated (U) probes (β = M/(M + U)). The β-values range from 0, indicating no methylation, to 1, indicating complete methylation. Differential methylation analysis was conducted using the Genome Studio Differential Methylation Analysis module across the entire set of CpG sites, which were annotated with their corresponding gene names. Genes were considered significant based on the DiffScores (>13 or <−13) and Delta beta (Δβ) values (>0.2 or <−0.2). An increased DiffScore denotes a larger difference in methylation between the two groups, suggesting that the probe may be subject to differential methylation and could potentially be associated with the biological condition being analyzed. Delta beta (Δβ) values exceeding 0.2 were classified as hypermethylated, while those below −0.2 were classified as hypomethylated. The EPIC v2 array manifest file was employed to annotate all differentially methylated probes (DMPs) with a *p*-value below the 0.05 threshold [51].

### 4.7. Proteomics

#### 4.7.1. RapiGest™ Protein Extraction

Cells (3 × 10^5^) were grown in six-well plates, and after treatment with ASMs (monotherapy and in combination with folic acid) for 24 h, cells were once washed with ice-cold phosphate-buffered saline (PBS). The cells were collected by scrapping in ice-cold PBS and were centrifuged at 2000 rpm for 5 min. The resulting cell pellet was resuspended in 50 µL RapiGest™ Protein Extraction buffer (0.5% RapiGest™ in 50 mM ammonium bicarbonate buffer) and sonicated for nucleic acid fragmentation. The cell lysis was further supported by subsequent freezing (liquid N_2_) and thawing (4 °C for 3 min) of the cells two times. The cell debris was pelleted by ultracentrifugation (10 min, 4 °C, and 12,000 rpm). Finally, we collected the protein-containing supernatants in new tubes and stored them at −80 °C until further use. Protein concentration was quantified by Bradford assay with bovine serum albumin as the standard protein. The desalting of all the samples was performed using Amicon Ultra 0.5 mL centrifugal filters with 3000 molecular weight cut-off (MWCO) membrane (Millipore, Burlington, MA, USA, UFC500324, 24Pk) as per manufacturer’s instructions.

#### 4.7.2. Protein Identification by Mass Spectrometry (The Nano LC-MS/MS)

Lysates were separated using SDS-PAGE and the bands were digested with trypsin. The resulting peptides were analyzed using an LC-MS/MS UltiMate™ 3000 RSCLC nano Ultra High-Performance Liquid Chromatography (UHPLC) system (Thermo Fisher Scientific, Waltham, MA, USA) paired with an Orbitrap Eclipse Tribrid mass spectrometer (Thermo Fisher Scientific, Waltham, MA, USA). Chromatographic separation took place on an EASY-Spray PepMap RSLC C18 column (100 Å 2 µm, 75 µm × 500 mm; Part No. ES903, Thermo Fisher Scientific, Waltham, MA, USA) maintained at 40 °C. A total of 300 ng of peptide from each sample was loaded onto the trapping column and separated at a flow rate of 250 nL/min using a binary gradient composed of Solvent A (0.1% (*v*/*v*) formic acid in LC-MS grade water) and Solvent B (0.1% (*v*/*v*) formic acid in acetonitrile). The gradient was programmed as follows: 5% B for 5 min, increase to 45% B over 90 min (up to 95 min), increase to 60% B by 110 min, then to 95% B by 120 min, hold at 95% B until 130 min, and return to 5% B at 131 min. The mass spectrometer operated in positive ion mode, equipped with a nano-spray ionization (NSI) source, with the capillary voltage set to 1400 V. A false discovery rate (FDR) of 0.01 (strict confidence) was used for peptide spectrum match (PSM) validation, and proteins were identified based on the presence of at least one unique peptide. Carbamidomethylation was designated as a static modification, while oxidation at methionine and phosphorylation at serine, threonine, and tyrosine were considered dynamic modifications. Data processing and analysis were performed using Proteome Discoverer software, version 3.0.1.27 (Thermo Fisher Scientific, Waltham, MA, USA).

#### 4.7.3. Data Processing and Differential Expression Analysis

The Mass Spectrometry Elevated Collision Energy (MS^E^) spectra obtained were processed and analyzed using Proteome Discoverer version 3.0.1.27 (Thermo Fisher Scientific, Waltham, MA, USA) to identify and quantify proteins. The data processing involved applying lock mass correction after acquisition to improve accuracy. Noise reduction thresholds were set at 150 counts for low-energy scan ions and 30 counts for high-energy scan ions to enhance data quality. Peptide identification was carried out through database searches against the human proteome repository from UniProt, using a strict false discovery rate (FDR) threshold of ≤1.0% to ensure reliable validation during the analysis. The parameters for protein identification required at least one fragment ion match per peptide, three fragment ion matches per protein, and a minimum of one peptide match for confident protein identification. Datasets were analyzed using the Hi-N algorithm, which resolves peptide conflicts by averaging the intensities of the three most abundant unique peptides linked to each protein. After identifying peptides and proteins, peptide abundance was calculated based on the total intensity of all constituent peptide ions, while protein abundance was determined by averaging the intensities of the most abundant peptides, providing a representative signal for each protein. Peptide abundance ranking was performed based on integrated values across all runs, aided by accurate alignment and the absence of missing data. This thorough methodology ensures robust peptide selection and supports reliable relative quantification of proteins across multiple analytical runs.

### 4.8. Databases and Resources for Pathway Analysis

Gene set enrichment analysis (GSEA) for functional gene annotation was performed using the gene ontology (GO) online tool [52]. GO annotation datasets for biological processes, molecular functions, and cellular components were analyzed using Fisher’s exact test and corrected for false discovery rate (FDR). Pathways with an FDR of less than 0.05 were considered statistically significant. To compare the datasets and identify overlaps, InteractiVenn, a web-based tool, was employed (https://www.interactivenn.net accessed on 15 January 2025) [53]. Upset plots were constructed using the UpSetR package 1.4.0 in R to delineate the intersection patterns of differentially methylated probes and differentially expressed proteins across various treatment conditions. Volcano plots, created with the ggplot2 package 3.5.2 in R, integrated abundance ratio and adjusted *p*-values to highlight significantly up- and downregulated proteins.

### 4.9. Statistical Analysis

The qRT-PCR findings were presented as the mean ± standard error, based on three independent experiments. Statistical significance among the groups was evaluated using one-way ANOVA in conjunction with Sidak’s multiple comparison test, where a *p*-value of less than 0.05 was considered significant. These statistical analyses were conducted using Gaphpad Prism software version 8.1. Differential methylation across the genome-wide methylation array was analyzed by comparing the condition group with the reference group using the Illumina Custom Model, the Mann–Whitney Model, and the T-Test Model. *p*-values were corrected for multiple comparisons using the Benjamini–Hochberg method, with a significance threshold set at *p* < 0.05. The LC-MS/MS analysis was performed with three independent biological replicates, each having two technical replicates. Protein abundance ratios were calculated as control relative to treated (i.e., Control/Treated). An abundance ratio > 1 indicated downregulation, whereas a ratio < 1 denoted upregulation in the treated condition compared to control. Proteomics data were processed and analyzed using the Proteome Discoverer software, version 3.0.1.27 (Thermo Fisher Scientific, Waltham, MA, USA). For subsequent gene enrichment analysis, only proteins meeting the criteria of an adjusted *p*-value less than 0.05 and a unique peptide count of ≥2 were included.

## 5. Conclusions

This study provides an integrated assessment of the epigenetic impact of first-generation antiseizure medications (ASMs) and folic acid (FA) on genes regulating DNA methylation, global methylation dynamics, and downstream proteomic alterations. Our findings demonstrate that ASMs can disrupt key developmental pathways through gene- and region-specific epigenetic modifications, while FA co-supplementation offers a protective effect by partially restoring methylation and protein expression profiles. The identification of drug-specific epigenetic signatures enhances our understanding of ASM-induced teratogenicity and may inform safer therapeutic strategies, particularly given the widespread use of these drugs across various clinical indications.

## Data Availability

All data pertaining to the work is included in the manuscript and supplementary files. The data is available from Xenod https://zenodo.org/records/15721137. Accessed on 23 June 2025.

## Supplementary Materials

The following supporting information is available at Xenod https://zenodo.org/records/15721137 (accessed on 23 June 2025).
